# The Seabury Vulcanizer—A Few Cautionary Suggestions in Its Use

**Published:** 1892-08

**Authors:** William H. Trueman

**Affiliations:** Philadelphia


### THE SEABURY VULCANIZER—A FEW CAUTIONARY SUGGESTIONS IN ITS USE.

BY WILLIAM H. TRUEMAN, D.D.S., PHILADELPHIA.

My purpose in writing is to relate, for the benefit of others, a little adverse experience in the use of the Seabury vulcanizer, I have had within the last few months from unexpected and unlooked-for causes, that might, with a machine less closely watched, have been disastrous; causes, however, readily avoided if the probability of their occurrence is known.

A few months ago, after several years' perfectly satisfactory experience with this machine, I was annoyed by plates being occasionally "under done," more frequently "over done," and in a few instances completely ruined by excessive heat. A steam-gauge that accurately indicated the pressure, and a very sensitive gas regulator that for some years has proved quite reliable, was, on examination, found to be in good condition, and should have rendered this quite impossible. All seemed in working order, and yet something was surely wrong. On taking the apparatus apart, the bent pipe connecting the gauge and regulator with the steam boiler was found almost completely closed with scale and rust, an exceedingly dangerous condition, indeed, one similar has been the cause of numerous disastrous steam-boiler explosions. The closure was not quite complete; under varying conditions the passage evidently was more or less open. At all times the gauge indicated steam pressure, but, owing to the obstruction, not accurately and not promptly. The condition of some plates showed plainly that the temperature of the vulcanizing chamber had been far in excess of that indicated by the recorded steam pressure. The fact that some plates came out properly vulcanized, and that others were under done, while the steam-gauge indicated the same pressure on each occasion, was sufficient evidence that this obstruction was not constant; that it at times prevented the steam pressure from reaching the gauge promptly, while on other occasions, doubtless a passage had been opened, perhaps suddenly, actuating the regulator and partially shutting off the gas-supply; the obstruction afterwards acting as a valve, had held the gauge at that point until the heat in the vulcanizer had been considerably reduced, thus accounting for the under-done plates. This erratic behavior of the vulcanizer baffled all efforts to discover the cause for some time; indeed, it was not suspected until, satisfied that something was wrong, the machine was taken apart.

The cause of annoyance and danger may be avoided by occasionally inspecting this fitting; with a small pair of gas-fitter's tongs it can be readily removed. Other vulcanizers than the Seabury have a similar fitting, connecting to them a steam-gauge. It will be well for those who use them to bear in mind the possibility that this passage, usually of small diameter and purposely made tortuous to prevent the steam coming in direct contact with the interior of the gauge, and in use filled with water that is liable to be charged with agents that may corrode either iron or brass, may be thus closed and become a source of serious danger. In using the Seabury vulcanizer, see that the gauge promptly responds when steam is admitted to the vulcanizing chamber, or when the escape-cock is momentarily opened. Take no risks with any vulcanizer; they are all strongly made, and will bear a much greater pressure than the work requires; on that account an explosion from over-pressure is more dangerous.

There is another source of annoyance and possible danger to which I desire to call attention. The Seabury vulcanizer in use (and I presume most if not all two-chambered vulcanizers, or those using dry or superheated steam) has a separate heat-supply for the boiler or steam-generator and for the vulcanizing chamber. When gas is used, both are controlled by the same cock and the same heat-regulator, which in all vulcanizers I have seen fitted with one is actuated by the steam pressure in the boiler. I see no objection to this arrangement, neither can I suggest any change sufficiently free from complications for practical every-day use that would prevent the difficulty to which I now refer. With a free, unobstructed passage between the two chambers it would seem that the heat and pressure in each should be precisely the same,—the pressure undoubtedly is; the heat, however, I have found by costly experience, may be far greater in the vulcanizing chamber than accords with the pressure of steam in the boiler.

This has occurred from two ascertained and preventable causes; there may be others as yet undiscovered.

*First.* It seems that when adding water to the boiler from time to time a little was spilled, and, running down the side of the boiler and across its bottom, dropped down exactly over the gas-burner of that side, and so corroded it that the gas-exit was reduced in size, and the gas flame thereby made much smaller than the one under the vulcanizing chamber. Be it understood that, in using the Seabury vulcanizer, when preparing to vulcanize, the cock controlling the passage between the two chambers is closed and kept closed until the steam-gauge indicates the desired pressure. The case is then placed in the vulcanizing chamber, the machine made steam-tight, and, lastly, the cock is opened admitting steam into the vulcanizing chamber. Both gas-jets are, however, in the beginning lighted at the same time, the intention being that, when the steam pressure in the boiler reaches the desired point, the other chamber shall be so heated that when steam is admitted to it it is not condensed. During the process of vulcanizing both jets are kept burning, it being presumed that, with an equal distribution of gas to each burner, the same amount of heat required to keep the steam in the boiler at the desired pressure will also keep the vulcanizing chamber at the required temperature. It seems to be essential to the proper working of the vulcanizer that within narrow limits the boiler and the vulcanizing chamber should each receive about the same amount of heat. The accidental corrosion of the burner under the boiler destroyed this equilibrium, and had resulted, before it was discovered, in the flame under the vulcanizing chamber, during the process of vulcanizing, being about twice as large as that under the boiler. When the gas was first lighted, as the two burners did not consume as much gas as would pass through the rubber tube connecting them with the gas-supply, the difference in the size of the flame was less noticeable than after the desired pressure was reached, and the gas-regulator, by partially closing the cock, reduced the gas pressure; the unobstructed burner then received much more than its share. The gas-regulator in use had for so long a time worked with such perfect satisfaction that the gas-jets were seldom inspected after they were lighted, and, consequently, this source of danger was for a long time unnoticed. Not until several plates were completely ruined by excessive heat was it discovered. Until then it was presumed that the steam-gauge indicated the precise temperature of both chambers, and that a greater degree of heat in either chamber than that corresponding to the indicated steam pressure could not be. The excessive heat to the flask and its contents was not due to direct contact with the bottom of the chamber, the flask in all cases being supported by a casting ring, and not in contact with the bottom or the walls of the chamber at any point.

Much the same condition of affairs may result if the vulcanizer leaks steam. More heat will then be required to keep up the steam pressure, and unless each source of heat is separately controlled, an arrangement not to be recommended, excessive heat will be communicated to the vulcanizing chamber and the work correspondingly impaired. Injury to the work is, however, the least important consideration. All heat applied to a vulcanizer, unregistered and unrecorded, is a serious source of danger. A broken thermometer, an unreliable steam-gauge or gas-regulator, the heat absorbed by dry steam, and that does not increase the pressure and thereby effect the steam-gauge, increases the risk attending this operation. If this excessive latent heat does not increase the pressure, it decreases rapidly the tenacity of the metal, and thereby weakens the walls of the vulcanizer. Or again, if from any cause a few drops of water should pass from the boiler into this superheated vulcanizing chamber, a mishap liable to occur from various causes, a destructive explosion would be inevitable.

In conclusion, this experience seems to teach that an excellent form of vulcanizer, fitted with perfectly reliable regulating and safety appliances, should be at all times, and at all points, closely watched and inspected, and occasionally overhauled.

Notwithstanding the unpleasant experience above related, so satisfactory has been our seven years' practical use of the Seabury vulcanizer that I would not willingly go back to the older form. We have found it so much more cleanly to use, so much more convenient for both packing and vulcanizing, and the resulting plates so solid and uniform in texture, that we can readily excuse this, the first trouble we have had with it, especially so since the cause being known it is not likely to again occur.

